# Alterations in choroidal vascular structures due to serum levels of vascular endothelial growth factor in patients with POEMS syndrome

**DOI:** 10.1038/s41598-023-37727-4

**Published:** 2023-06-30

**Authors:** Hirotaka Yokouchi, Daisuke Nagasato, Yoshinori Mitamura, Mariko Egawa, Hitoshi Tabuchi, Sonoko Misawa, Satoshi Kuwabara, Takayuki Baba

**Affiliations:** 1 Department of Ophthalmology and Visual Science, Graduate School of Medicine, Chiba University, 1-7-1, Inohana, Chiba, 260-0856 Japan; 2 Department of Ophthalmology, Saneikai Tsukazaki Hospital, Himeji, Japan; 3 Department of Ophthalmology, Institute of Biomedical Sciences, Tokushima University Graduate School, Tokushima, Japan; 4 Department of Neurology, Graduate School of Medicine, Chiba University, Chiba, Japan

**Keywords:** Medical research, Diseases, Eye diseases, Retinal diseases

## Abstract

A higher serum vascular endothelial growth factor (VEGF) level can cause choroidal thickening in the choroid of patients with polyneuropathy, organomegaly, endocrinopathy, monoclonal gammopathy, and skin changes (POEMS) syndrome. We aimed to determine whether fluctuations in serum VEGF levels affect choroidal vascular structures in patients with POEMS syndrome. This retrospective observational case series examined 17 left eyes of 17 patients with POEMS syndrome. Enhanced depth imaging optical coherence tomography (EDI-OCT) images were obtained, and serum VEGF levels were measured at baseline and 6 months after transplantation with dexamethasone (n = 6), thalidomide (n = 8), or lenalidomide (n = 3). EDI-OCT images were binarized using ImageJ software, and we calculated the areas of the whole choroid and the luminal and stromal areas. Subsequently, we determined whether the choroidal vascular structure had changed significantly between baseline and 6 months after treatment. Six months after treatment, serum VEGF levels and the whole choroid, luminal, and stromal areas had decreased significantly compared to the baseline values (all, P < 0.001). The mean luminal area to the whole choroidal area ratio at 6 months after treatment was 0.70 ± 0.03, which was significantly smaller than the ratio at baseline (0.72 ± 0.03; P < 0.001). Whole choroid and luminal area fluctuations were significantly positively correlated with fluctuations in serum VEGF levels (r = 0.626, P = 0.007 and r = 0.585, P = 0.014, respectively). Choroidal thickening induced by VEGF might be caused by increases in the choroidal vessel lumen area. These results may offer insights into the pathogenesis of POEMS syndrome and the role of serum VEGF in choroidal vascular structure, which may apply to other ocular diseases.

## Introduction

Vascular endothelial growth factor (VEGF) is overproduced, which promotes vascular permeability and neovascularisation, in patients with polyneuropathy, organomegaly, endocrinopathy, monoclonal gammopathy, and skin changes (POEMS) syndrome^1,2^. These actions may cause the characteristic features of POEMS syndrome, including organomegaly, oedema, pleural effusion, ascites, and angiomata^2^. In many cases, optic disc oedema has been considered a major ocular finding in POEMS syndrome^1,3–5^. A standard treatment for POEMS syndrome has not been established, although some studies have reported that autologous peripheral blood stem cell transplantation, lenalidomide therapy, thalidomide therapy, and systemic anti-VEGF agents, such as bevacizumab, are effective^6–9^.

Recently, advances in imaging methods such as optical coherence tomography (OCT) can allow non-invasive choroidal evaluations. For example, Sonoda et al.^10^ have reported a new method for analysis of choroidal structures using binarised enhanced depth imaging OCT (EDI-OCT) images. Analysing these images can help evaluate choroidal thickness (CT) and component ratios such as the whole choroid, stromal, and luminal areas. Nagasato, a co-author of this study, has used this method to analyse choroidal structure and revealed novel finding about physiological changes in the choroid, including structural changes due to coffee and water intake^11,12^. Mitamura reported that changes in choroidal structure occur due to various retino-choroidal diseases and is associated with treatments and prognosis^13–15^.

Previously, we reported that patients with POEMS syndrome presented thickened choroids, and choroidal thinning was observed after treatment and a decrease in serum VEGF levels^5,16^. Based on these findings, we suggest that a higher serum VEGF level may cause changes in the choroid of patients with POEMS syndrome. However, the potential effects of serum VEGF levels on the ratio of stromal and luminal components in the choroid are unclear. Therefore, we aimed to analyse the choroidal vascular structure using binarised EDI-OCT images to confirm whether serum VEGF levels may influence the whole choroidal, stromal, and luminal areas in the choroid of patients with POEMS syndrome.

## Materials and methods

### Ethics statements

The Institutional Review Board of Chiba University (number: 3374) approved the protocol and design of this study. We conformed all procedures to the tenets of the Declaration of Helsinki, and all patients gave their informed consent to participate in this study after they were informed about its aims and potential complications.

### Study design and population

In this retrospective observational case series, we evaluated the 17 left eyes of 17 treatment-naïve Japanese patients with POEMS syndrome at the Chiba University Hospital visiting from January 2016 to June 2021. POEMS was diagnosed based on the criteria established by Kuwabara and Misawa^17^. Exclusion criteria involved patients whose eye showed any of the following findings: (1) a past and current history of glaucoma, intraocular surgery, choroidal vascular, and retinal disease; (2) a spherical equivalent (refractive error) > − 6.0 dioptres (D); (3) an axial length (AL) greater than 26.5 mm; and (4) an intraocular pressure (IOP) > 20 mmHg.

### Treatments for patients with POEMS syndrome

The patients were treated with transplantation (n = 6), thalidomide (n = 8), or lenalidomide (n = 3) with dexamethasone. We initiated thalidomide treatment at a dose of 100 mg daily systemically and increased the dose to 300 mg per day with dexamethasone (approximately 12 mg/m^2^), which was added for 4 days at intervals, once a month for 6 months^17^. Lenalidomide is a derivative of thalidomide, and its efficacy and safety in patients with POEMS syndrome have been reported^8^. The regimen was administered as six 28-day cycles with lenalidomide on days 1–21 (15 mg during cycle 1 and 25 mg during cycles 2–6) plus dexamethasone once a week (20 mg). We performed autologous peripheral blood stem cell collection after mobilisation using subcutaneous administration of the granulocyte colony-stimulating factor with or without high-dose cyclophosphamide (2 g/m^2^/day for two consecutive days). Following blood cell collection, we performed stem cell transplantation for approximately 1 month and high-dose melphalan chemotherapy (140–200 mg/m^2^). Subsequently, we reduced the dose of melphalan as the same protocol for patients with performance status 4 (completely disabled)^18^.

### EDI-OCT imaging

EDI-OCT images were obtained using spectral domain OCT (Heidelberg Spectralis OCT, Heidelberg, Germany). Details of the analysis of EDI-OCT images, including foveal thickness (FT), have been previously described^11–13^. We performed all OCT examinations between 12:00 PM and 3:00 PM to minimize the influence of diurnal variations in the choroid. Differences in the readings between the two observers were within 10% of the mean value. EDI-OCT images were evaluated within 1 or 2 weeks after we collected the patients’ blood samples at baseline and 6 months after treatment.

### Analysis of the choroidal structure

In our previous studies, we suggested a high degree of reproducibility on implementing analyses of the choroidal component ratio^11–13^. In previous reports, we have noted the details of binarisation techniques used to analyse choroidal structures^10,19,20^. We used the open-access ImageJ software package (version 1.47, National Institutes of Health, Bethesda, MD, USA), and each white-on-black EDI-OCT image was analysed using the Niblack method, which is a binarisation processing method in the software. Subsequently, we selected three low-luminance areas in the centre of a blood vessel with a diameter greater than 100 µm in the outer-choroid by using the Oval Selection Tool. To reduce the noise of the captured image, the mean reflectance of the three regions was calculated and used as the minimum image luminance. We manually set the choroidal area, which is located around the fovea as the analysis area, which was a 1500-μm-wide area between the chorio-scleral border and the Bruch membrane.

**Figure 1:**
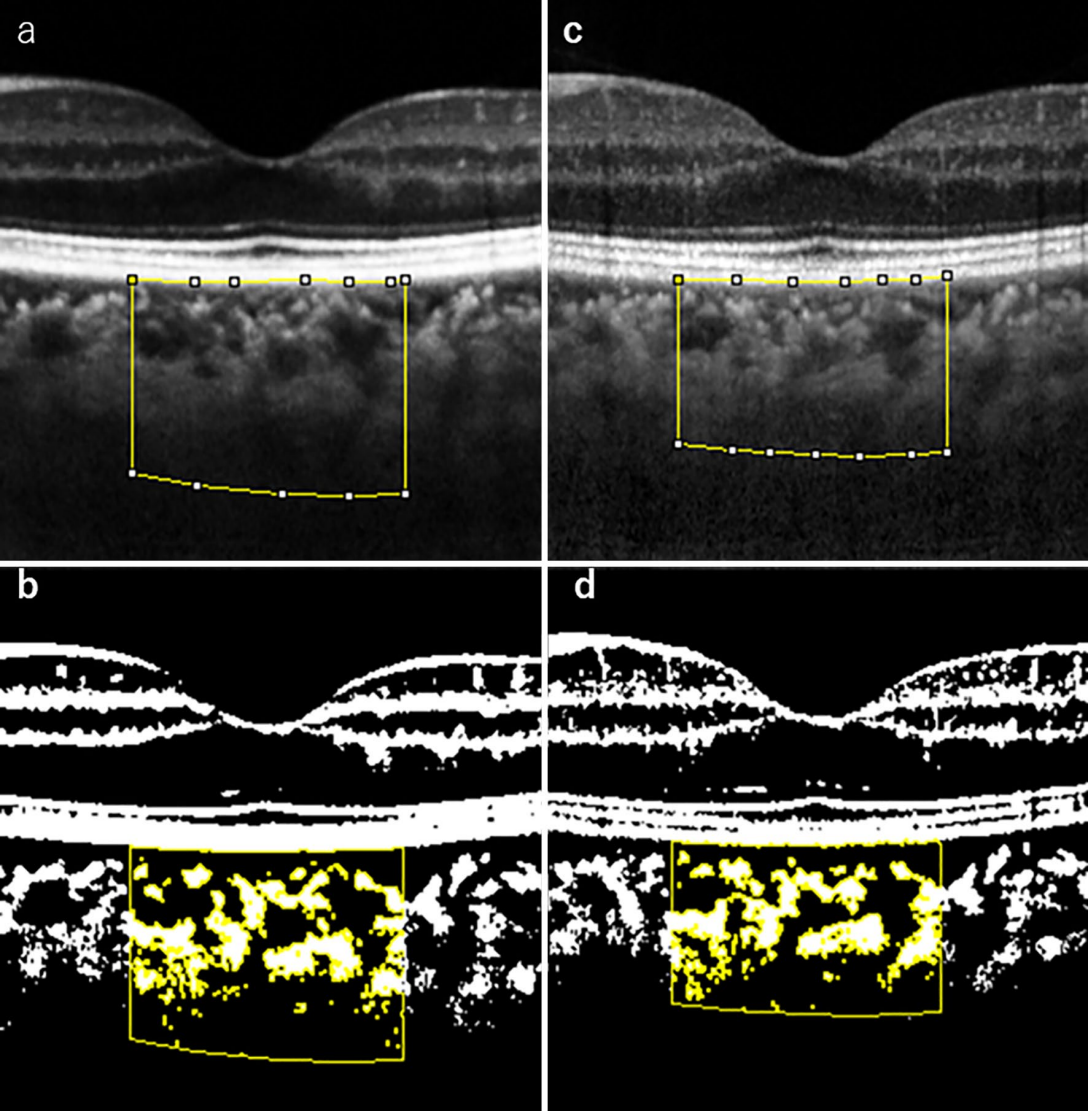
Choroidal structure analysis via enhanced depth imaging (EDI) optical coherence tomography (OCT). An EDI-OCT image was converted to a binary image using ImageJ software (Bethesda, MD). The luminal choroidal area and the stroma choroidal area are seen in this image. The rectangle surrounded by a red line was excised, and the dark areas were traced using the Niblack method. In this situation, EDI-OCT images were gained in the macular area via horizontal sectioning (the optic disc is localised on the left side). A 1500-μm area covering the fovea was segmented and analysed in this study. The whitish and blackish regions correspond to the stromal and luminal areas, respectively. EDI-OCT images (left eye) of the 42-year-old female patient with POEMS syndrome reported reveals a choroidal thickening at baseline (**a**,**b**) and choroidal thinning 6 months post-treatment (**c**,**d**). An EDI-OCT image was converted to a binary image using the Niblack method, available within ImageJ software. (**a**) EDI-OCT image of left eye at baseline (**b**) Converted binary image of EDI-OCT image of left eye at baseline (**c**) EDI-OCT image of left eye 6 months post-treatment (**d**) Converted binary image of EDI-OCT image of left eye 6 months post-treatment.

Using the function of Niblack Auto Local Threshold, we produced grayscale images, which can display 256 shade gradations and be automatically replaced with two black-white gradations. Therefore, we defined these areas with black and white pixels as the stromal and luminal areas, respectively. Subsequently, data on the relationship between the real fundus distance and spacing of the pixel from the EDI-OCT images were automatically calculated using ImageJ software (Fig. 1a–d).

Subsequently, we determined the whole choroidal, luminal, stromal areas, and the ratio of the luminal area to the whole choroidal area (the L/W ratio). These parameters were measured three times per EDI-OCT image, and the mean values of the three measurements were used in analyses. All clinical findings were masked during choroidal structure analysis. Details of measurement of the serum VEGF levels using enzyme-linked immunosorbent assay have been described in our previous reports^3,5,6,16^ (Quantikine HS®, R&D Systems, Minneapolis, MN, USA).

Additionally, systolic blood pressure (SBP), diastolic blood pressure (DBP), and heart rate (HR) were measured on the left arm using a commercial sphygmomanometer. We also measured intraocular pressure (IOP) in the left eye. Mean arterial pressure (MAP) and mean ocular perfusion pressure (MOPP) were calculated using the equations MAP = DBP + 1/3 (SBP–DBP) and MOPP = 2/3 MAP – IOP, similar to our previous report^11,12^.

### Statistical analyses

Using the Wilcoxon signed-rank test, we determined significant differences in the mean serum VEGF level, FT, whole choroidal, luminal, stromal areas, and the L/W ratio between baseline and 6 months post treatment. Additionally, we determined significant differences in SBP, DBP, HR, IOP, MAP, and MOPP between baseline and 6 months post treatment. The correlations between the fluctuations in serum VEGF levels and FT and the whole choroidal, luminal, and stromal areas were determined using Spearman’s rank correlation coefficients. We also determined the correlations between the fluctuations in serum VEGF levels and SBP, DBP, HR, IOP, MAP, and MOPP from baseline to 6 months post treatment.

We performed all statistical analyses using SPSS software for Microsoft Windows (Version 27, IBM, Tokyo, Japan) and defined significance as *P* < 0.05.

## Results

Seventeen left eyes of 17 treatment-naïve Japanese patients with POEMS syndrome (10 men and 7 women) were evaluated in this study. The patients’ mean age was 51.7 ± 14.1 years (range, 34–75 years; median 54 years), the mean spherical equivalent (refractive error) was − 1.01 ± 2.29 D (range, − 5.75 to + 3.25 D; median − 0.5 D), and the mean AL was 23.9 ± 1.01 mm (range, 22.72–26.35 mm; median 23.78 mm). The mean IOP was 11.7 ± 2.96 mmHg (range, 6–17 mmHg; median 12 mmHg).

At baseline, the mean (± standard deviation) SBP, DBP, and HR values were 128.3 ± 14.1 mmHg (range, 106–154 mmHg; median 127 mmHg), 73.6 ± 9.6 mmHg (range, 56–88 mmHg; median 72 mmHg), and 73.1 ± 11.1 mmHg (range, 54–100 mmHg; median 73 mmHg), respectively. The mean MAP and MOPP were 91.8 ± 10.3 (range, 73.3–109; median 91.3) and 49.4 ± 6.38 (range, 41.8–60.7; median 50.2), respectively (Table 1).

**Table 1:** The mean SBP, DBP, HR, IOP, MAP, MOPP and levels of mean serum VEGF levels before and after treatment.

	Baseline	6 months after treatment	p value
SBP (mmHg)	128.3 ± 14.1	127.8 ± 11.6	0.619
DBP (mmHg)	73.6 ± 9.67	72.2 ± 8.04	0.797
HR	73.1 ± 11.1	72.6 ± 10.0	0.798
IOP (mmHg)	11.7 ± 2.96	11.8 ± 2.91	0.773
MAP	91.8 ± 10.3	91.6 ± 7.80	0.918
MOPP	49.4 ± 6.38	49.3 ± 5.15	0.959
VEGF (pg/ml)	7010 ± 3314	574 ± 484	0.001

*SBP* systolic blood pressure, *DBP* diastolic blood pressure, *HR* heart rate, *IOP* intraocular pressure, *MAP* arterial pressure, *MOPP* ocular perfusion pressure, *VEGF* vascular endothelial growth factor.

MAP = DBP + 1/3(SBP – DBP), MOPP = 2/3 MAP – IOP.

Six months after treatment, the mean serum VEGF level in all patients significantly decreased to 574 ± 484 pg/mL (range, 164–1870 pg/mL; median 488 pg/mL) from the baseline 7010 ± 3314 pg/mL (range, 4090–16,400 pg/mL; median 6450 pg/mL) (P = 0.001; Table 1). The baseline mean serum VEGF level was approximately 30 times higher than that of normal individuals (220 pg/mL) (21).

After 6 months, the mean (± standard deviation) SBP, DBP, HR, and IOP were 127.8 ± 11.6 mmHg (range, 105–157 mmHg; median 130 mmHg), 72.2 ± 8.04 mmHg (range, 60–90 mmHg; median 72 mmHg), 72.6 ± 10.0 mmHg (range, 55–93 mmHg; median 72 mmHg), and 11.8 ± 2.9 mmHg (range, 6–16 mmHg; median 12 mmHg), respectively. The mean (± standard deviation) MAP and MOPP were 91.6 ± 7.8 (range, 79.6–106.6; median 90.6) and 49.3 ± 5.15 (range, 42.1- 59.1; median 50.2), respectively. These factors did not significantly change between baseline and after 6 months (*P* > 0.05) (Table 1).

At baseline, the mean whole choroid area was 55.5 × 10^4^ ± 10.6 × 10^3^ μm^2^ (range, 34.8 × 10^4^ to 71.8 × 10^4^ μm^2^; median 56.9 × 10^4^ μm^2^), the luminal area was 40.7 × 10^4^ ± 84.7 × 10^3^ μm^2^ (range, 24.5 × 10^4^ to 52.4 × 10^4^ μm^2^; median 43.2 × 10^4^ μm^2^), the stromal area was 14.9 × 10^4^ ± 29.0 × 10^3^ μm^2^ (range, 10.1 × 10^4^ to 19.7 × 10^4^ μm^2^; median 14.2 × 10^4^ μm^2^) and the L/W ratio was 0.72 ± 0.03 (range, 0.67 to 0.79; median 72.6).Six months after treatment, the mean whole choroid area decreased to 42.2 × 10^4^ ± 10.8 × 10^3^ μm^2^ (range, 24.7 × 10^4^ to 63.0 × 10^4^ μm^2^; median 45.0 × 10^4^ μm^2^), which was significantly smaller than the baseline mean whole choroidal area (*P* < 0.001; Table 2). The mean luminal area of 30.1 × 10^4^ ± 82.5 × 10^3^ μm^2^ (range, 16.5 × 10^4^ to 45.0 × 10^4^ μm^2^; median 30.5 × 10^4^ μm^2^) and the stromal area of 12.1 × 10^4^ ± 29.8 × 10^3^ μm^2^ (range, 6.8 × 10^4^ to 17.9 × 10^4^ μm^2^; median 12.5 × 10^4^ μm^2^) also decreased significantly compared to the baseline values (both, *P* < 0.001, Table 2). The mean L/W ratio was 0.70 ± 0.03 (range, 0.65 to 0.79; median 70.8) at 6 months after treatment, and it was significantly smaller than that at baseline (*P* < 0.001; Table 2). On the other hand, the mean FT did not significantly change (*P* > 0.05).

**Table 2:** The mean whole, luminal, stromal choroidal area, foveal thickness and levels of mean serum VEGF levels at baseline and after 6 months post-treatment.

	Baseline	Six months post-treatment	p value
Foveal thickness (FT) (μm)	221.1 ± 29.1	213.5 ± 15.8	NS
Whole choroidal area (W)(μm2)	55.5 × 104 ± 10.6 × 103	42.2 × 104 ± 10.8 × 103	< 0.001
Luminal choroidal area (L) (μm2)	40.7 × 104 ± 84.7 × 103	30.1 × 104 ± 82.5 × 103	< 0.001
Stromal choroidal area (S) (μm2)	14.9 × 104 ± 29.0 × 103	12.1 × 104 ± 29.8 × 103	< 0.001
L/W ratio	0.72 ± 0.03	0.70 ± 0.03	< 0.001
VEGF (pg/ml)	7010 ± 3314	574 ± 484	0.001

*VEGF* vascular endothelial growth factor, *L/W ratio* ratio of luminal area to the whole choroidal area.

**Figure 2:**
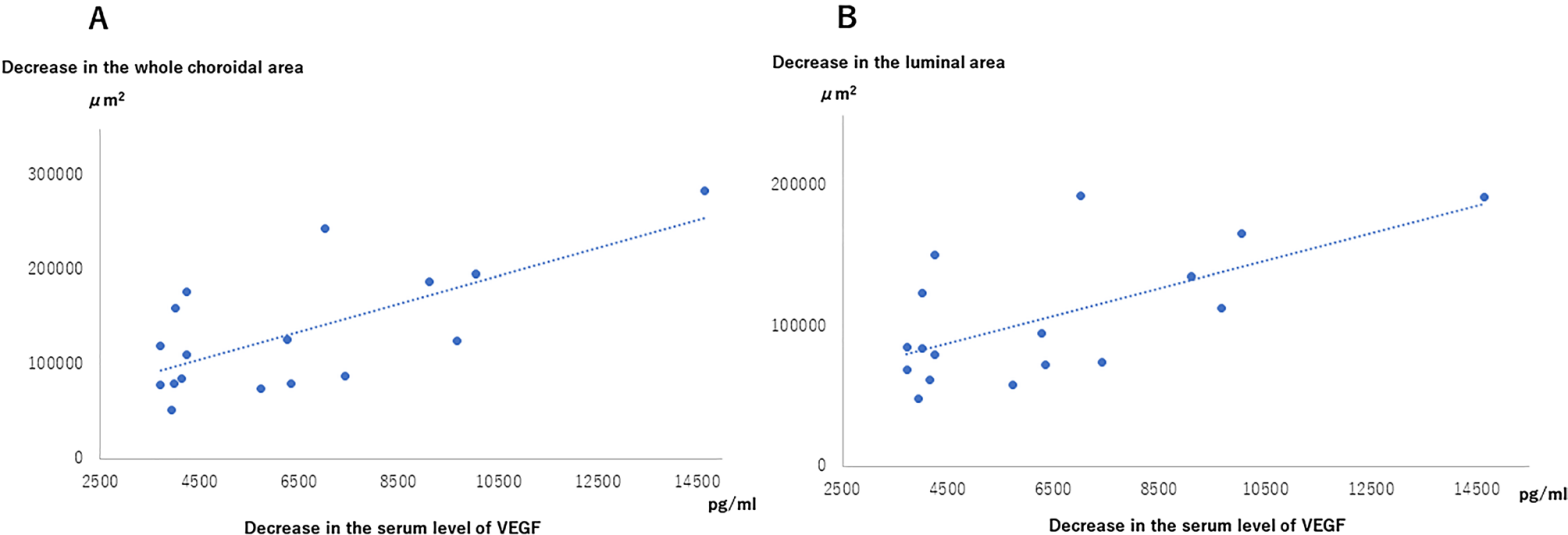
Correlations between fluctuations in whole choroidal and luminal areas and in serum VEGF (vascular endothelial growth factor). (**A**) Correlation between fluctuations in the whole choroidal area and in serum VEGF levels in the left eye. The whole choroidal area significantly decreased along with serum VEGF levels (r = 0.626, P = 0.007, Spearman’s rank correlation coefficient). (**B**) Correlations between fluctuations in the luminal area and in the serum VEGF levels in the left eye. The luminal choroidal area was decreased significantly along with a reduction in serum VEGF levels, and the correlation between fluctuations in the luminal area and in serum VEGF levels in patients with POEMS syndrome was significant (r = 0.585, P = 0.014, Spearman’s rank correlation coefficient).

Fluctuations in the whole choroidal area and the luminal area from baseline linearly and significantly correlated with the fluctuations in the baseline serum VEGF level (r = 0.626, *P* = 0.007 and r = 0.585, *P* = 0.014, respectively) (Fig. 2A and B). However, the fluctuation in the stromal area from baseline did not significantly correlate with the fluctuation in the baseline serum VEGF level (*P* > 0.05).

Fluctuations in SBP, DBP, HR, IOP, MAP, and MOPP from baseline were not significantly correlated with fluctuations in the serum VEGF level from baseline (*P* > 0.05).

**Figure 3:**
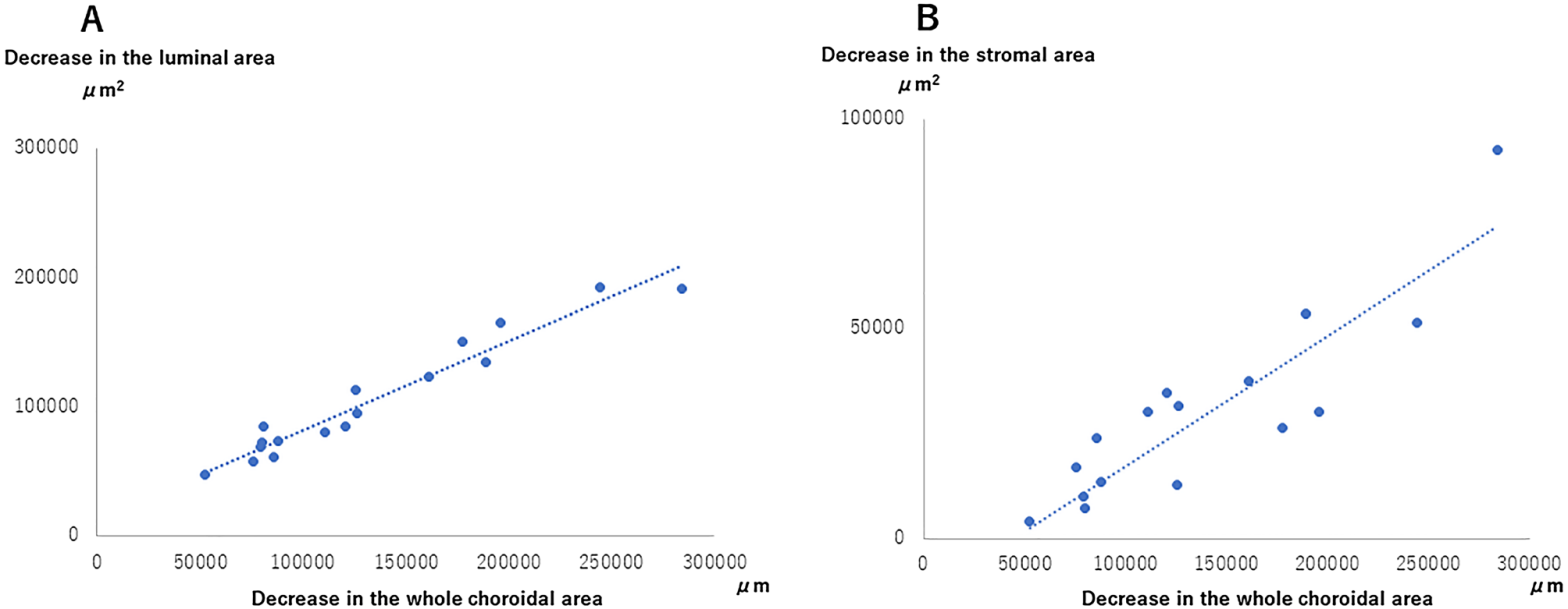
Correlations between fluctuations in luminal and stroma areas and in whole choroidal area. (**A**) Correlations between fluctuations in luminal area and in the whole choroidal area. There was a significant correlation between fluctuations in luminal area and in whole choroidal area in patients with POEMS syndrome (r = 0.963, P < 0.001, Spearman’s rank correlation coefficient). (**B**) Correlations between fluctuations in the stromal area and in the whole choroidal area. There was a significant correlation between fluctuations in luminal area and in the whole choroidal area in patients with POEMS syndrome (r = 0.797, P < 0.001, Spearman’s rank correlation coefficient).

Additionally, fluctuations in the luminal and stroma area were significantly and linearly correlated with fluctuations in the whole choroidal area from baseline to 6 months (r = 0.963, *P* < 0.001 and r = 0.797, *P* < 0.001, respectively) (Fig. 3A and B).

Fluctuations in SBP, DBP, HR, IOP, MAP, and MOPP from baseline were not significantly correlated with fluctuations in the whole choroidal area from baseline (*P* > 0.05).

No serious adverse effects were observed in these case series, including lenalidomide or thalidomide neuropathy, during or after these treatments. Additionally, no toxic death or serious adverse effects occurred during stem cell mobilisation and autologous peripheral blood stem cell transplantation.

## Discussion

In this study, fluctuations in the whole choroid area and luminal area from baseline significantly correlated with fluctuations in the serum VEGF levels from baseline, whereas no significant fluctuations were observed in stromal area and FT. The L/W ratio was 72.5% at baseline and 70.6% 6 months after treatment. Fluctuations in the luminal area and stromal area from baseline correlated linearly and significantly with fluctuations in the whole choroidal area from baseline.

Several factors can affect CT, including age, refractive error^21^, IOP, and AL^22^. In highly myopic eyes, the choroid is very thin and further thins with ageing and with an increase in the degree of myopia^21^. In our patients, we did not observe any significant differences in age, refractive error, IOP, and AL between baseline and 6 months after treatment. Although our sample size was small, we believe that the impact of age, IOP, and AL on whole choroidal area was likely minimal.

Regarding the specific components of the choroid, the L/W ratio significantly decreased from 72.5 at baseline to 70.6% after treatment. Previous studies have reported that MOPP and MAP have important effects on the choroidal component^11,12^. In our study, even though patients showed significantly reduced L/W ratios, their SBP, DBP, HR, and IOP remained unchanged (P > 0.05). Additionally, MAP and MOPP also remained unchanged after 6 months (P > 0.05).

These findings suggest that the reported changes in choroidal structure following treatment may be associated with a reduction in luminal area. While previous reports have indicated that most POEMS patients have a thickened choroid^5,16^, the specific details regarding the stromal and luminal components in the choroid are not well known. Thus, our study reveals that the expansion of choroidal luminal vessels is also notably feature in this disease.

VEGF osmotically enables intravascular active molecules to enter the stromal area resulting in stromal tissue swelling. Moreover, VEGF receptor 2 is the major mediator of vascular permeability and has an effect on enhancing diameter^23–26^. We demonstrated that the decrease in the whole choroidal area observed in this study was because of the contraction of the luminal and stromal regions. In addition, we speculated that serum VEGF can make choroidal blood vessels leak, resulting in oedema of the choroidal stroma and expansion of the diameter of choroidal vessels. Regarding the expansion of the diameter of choroidal vessels, VEGF can dilate vessels by upregulating endothelial nitric oxide synthase-dependent pathways^27–29^. For example, VEGF increased the vessel diameter in experimental animals^30^. In humans, anti-VEGF therapy decreased the choroidal luminal area in eyes with diabetic macular oedema^31^ and polypoidal choroidal vasculopathy^32^. However, the mechanism by which serum VEGF levels contribute to altering the choroidal luminal and stromal areas has not been fully understood.

We speculated that alterations in the choroid might occur due to an increase in choroidal vascular permeability and an expansion of the diameter of choroidal vessels caused by higher serum VEGF levels. In contrast, these therapies and decreased serum VEGF levels did not affect the fluctuation in FT. That might be why FT is probably affected by the location of VEGF receptors, especially VEGF receptors 1 and 2, which have different apico-basal distributions. Unlike luminal VEGF, abluminal VEGF might increase permeability and dilate vessels^33^. A high concentration of VEGF in the plasma leaks into the choroidal stroma area through the fenestrated choriocapillaris without a barrier function where some materials can freely leak. Concerning permeability and expansion of the diameter of the choroid capillaries, vascular endothelial cells in the choroid are abluminally affected by VEGF. In contrast, in the retina, vascular endothelial cells are luminally affected by VEGF; therefore, the permeability and expansion of the diameter of retinal capillaries should not occur.

This study had several limitations. First, we could not exclude the influence of other factors such as systemic and local medications, nutrition, and inflammatory cytokines (e.g. interleukin 6) that may contribute to changes in choroidal structure. Our patients were treated through transplantation (n = 6), thalidomide (n = 8), or lenalidomide (n = 3), along with dexamethasone. Among these three treatment groups, we did not observe a significant change in serum VEGF levels (p = 0.16), whole choroidal area (p = 0.43), luminal area (p = 0.37), stromal area (p = 0.58), MAP (p = 0.44), and MOPP (p = 0.44) between baseline and 6 months post treatment. Despite the limited sample size of only 17 patients, we believed that these treatments had minimal effects on choroidal thickness in our patients.

Second, we could not consider our results directly as a cause–effect relationship between higher levels of serum VEGF and the choroid structure because we could not directly examine the intraocular VEGF concentration in the choroid. Third, we could perform a binarisation technique-based analysis automatically; however, we ended up manually setting these choroidal areas to analyse them. An objective evaluation method that can automatically detect the chorio-scleral borders of the Bruch membrane is needed. Fourth, we only analysed these choroidal structure changes in the narrow range of 1500 μm, which was centred on the fovea. Therefore, we need to perform analysis in a larger choroidal area with similar changes occurring across the whole choroid. Fifth, we need angiographic examinations, such as indocyanine green and fluorescein angiography, to confirm vascular permeability in the choroid caused by high serum VEGF. However, we could not obtain these angiographic studies from our patients because of the poor systemic condition of most of the patients with oedema elsewhere in their body. Sixth, the sample size in this study is limited due to the rarity of the disease, and the small number of participants (only 17 patients) does not provide strong evidence to support the study’s conclusion. In fact, a Japanese national survey conducted in 2003 showed that its prevalence is 0.3/100 000 population^34^. Considering the limitations, we cannot definitively conclude that solely reducing serum VEGF levels will have an impact on the choroidal luminal area. As POEMS syndrome is very rare, we designed the protocol to include as many patients as possible based on the available cases. Thus, this study was designed and initiated without calculating a sample size that could determine conclusive efficacy. Further studies with larger sample sizes are needed to better understand the role of elevated serum VEGF in the changes occurring in choroidal structure.

In conclusion, our findings revealed a significant decrease in whole choroidal, luminal, and stromal areas after 6 months of treatment. Fluctuations in serum VEGF levels were significantly correlated with those in whole choroidal and luminal areas. We concluded that VEGF-induced choroidal thickening may be attributed to an increase in the choroidal vessel lumen area, with the expansion of choroidal luminal vessels being feature in this disease. The association between choroidal parameters and higher serum VEGF levels may provide insights into the pathogenesis of ocular diseases with POEMS syndrome and the role of serum VEGF in the choroidal dynamics in the context of other ocular conditions.

## Data Availability

The datasets used and/or analysed during the current study available from the corresponding author on reasonable request.
